# Reconceptualizing cybersecurity awareness capability in the data-driven digital economy

**DOI:** 10.1007/s10479-022-04844-8

**Published:** 2022-08-02

**Authors:** Shahriar Akter, Mohammad Rajib Uddin, Shahriar Sajib, Wai Jin Thomas Lee, Katina Michael, Mohammad Alamgir Hossain

**Affiliations:** 1 School of Business, University of Wollongong, Wollongong, NSW 2522 Australia; 2 School of Business, University of Wollongong, NSW 2522 Wollongong, Australia; 3 UTS Business School, University of Technology Sydney, 15 Broadway, Ultimo, NSW 2007 Australia; 4 School for the Future of Innovation in Society, School of Computing and Augmented Intelligence, Arizona State University, Mailcode 85287 Tempe, USA; 5 School of Accounting, Information Systems, and Supply Chain, RMIT University, Melbourne, VIC 3000 Australia

## Abstract

Data breaches have become a formidable challenge for business operations in the twenty-first century. The emergence of big data in the ever-growing digital economy has created the necessity to secure critical organizational information. The lack of cybersecurity awareness exposes organizations to potential cyber threats. Thus, this research aims to identify the various dimensions of cybersecurity awareness capabilities. Drawing on the dynamic capabilities framework, the findings of the study show personnel (knowledge, attitude and learning), management (training, culture and strategic orientation) and infrastructure capabilities (technology and data governance) as thematic dimensions to tackle cybersecurity awareness challenges.

## Introduction

Threats of cybercrimes have created tremendous challenges for organizations in today’s data-driven digital economy (Rawat et al., 2019). The seriousness of the issue of cybersecurity has compelled managers and policymakers to reevaluate cybersecurity measures at the individual, organizational, sectoral and national levels (Al-Shanfari et al., 2020; Schneider et al., 2020; Bauer et al., 2017; Zwiling et al., 2020; Granåsen & Andersson, 2016). Research on cybersecurity has a multidisciplinary orientation with important research focusing on technological, infrastructural, sociotechnical, psychological and educational aspects (Alotaibi et al., 2016; Bavel et al., 2019; Trim & Lee, 2019; Tschakert & Ngamsuriyaroj, 2019). While technology plays a critical role in tackling cybersecurity issues, more recently human aspects have gained serious attention (Michael, 2008; David et al., 2020; Maalem Lahcen et al. 2020). Specifically, the importance of specialized training, education, and knowledge of cybersecurity for individual employees, coupled with critical management capabilities and infrastructure, has been emphasized by scholars and practitioners. These aspects are seen as most essential in building cybersecurity awareness within and across organizational boundaries in the current data-driven business environment (Zwiling et al., 2020; Alotaibi et al., 2016; Holdworth & Apeh, 2017; Al-Janabi & Al-Shourbaji, 2016).

Due to the emergence of big data and data-driven business processes and operations in the present business environment, securing, protecting and defending organizational information has become more important than ever (Rawat et al., 2019; Granåsen & Andersson, 2016). In the latest report of the Australian Cybersecurity Centre for the 2020–2021 fiscal year, a total of 67,500 cybercrimes were reported. That is an increase of 13 percent year-over-year (Brown, 2021). It is estimated that the cost of cyber-crimes will reach 10.5 trillion USD by the year 2025 (CS Ventures, 2016). In 2020, losses from cyber-related crimes totalled AUD 33 billion in Australia (Brown, 2021). Further, a recent report prepared by the Office of the Australian Information Commissioner (2021) said that 61% of notifiable data breaches that occurred in the first half of 2020 were malicious or criminal attacks, an increase of 47% year-over-year. In fact, the year 2020 has experienced a record number of cyber-attacks on business enterprises, governments and individuals. Among these, there has been a significant increase in attacks related to interconnected emerging technologies, such as artificial intelligence, machine learning and 5G networks (Zhang et al., 2021). Attacks in 2020 are also showing greater tactical cooperation between hacker groups and state actors (Brooks, 2021).

The alarming increase in the rate of cyber attacks has led government and law enforcement to prioritize the exploration of effective methods of creating cybersecurity awareness in organizations to safeguard critical assets and infrastructure from cyber threats. The Australian Government has committed to investing $1.35 billion in cyber defence funding and has urged all Australians to strengthen their cyber defences. But experts warn such government measures will have limited impact unless businesses also take more effective action, internally, to educate their employees to improve cybersecurity (Proofpoint, 2021). These circumstances of increased attacks, government commitment to action, and concerns about the necessity for businesses necessitate to address cybersecurity issues have led to general increased awareness and to the issues of cyber threats and cybercrimes becoming a serious agenda for researchers and practitioners (Karjalainen et al., 2020).

As more and more attackers are now targeting people in organizations with limited knowledge of cybersecurity, updating the technological side of the cybersecurity system will not benefit an organization if the people working in the system are ignored. Therefore, it is vital to improve workforce security awareness by promoting cybersecurity knowledge and teaching practical approaches for responding to threats (Ani et al., 2019). An additional factor affecting organizations in the current situation is that the COVID-19 pandemic has created significant shifts in the way organizations operate. Many employees are working remotely with flexible working conditions, away from physically secured office buildings and clearly defined and protected systems perimeters. As a result, an organization’s technology environment can be more vulnerable to phishing attacks and other cybersecurity threats, such as social engineering (Brooks, 2021; Brown, 2021). Moreover, the working population has increasingly adopted mobile technology through bring your own device (BYOD) incentives, and in general, lacks fundamental knowledge of cybersecurity procedures for the protection of organizational assets and data. Employees also fall short in awareness of all the various threat vectors that are continually changing the corporate security landscape (Zwilling et al., 2020). In a recent survey, it was revealed that 45% of working adults reuse passwords, only 49% confirm password protection of their home Wi-Fi network, 26% consider a free Wi-Fi network of a trusted location as safe, and 17% are not sure about the safety of open access networks (Proofpoint, 2021). The importance of internal operational aspects for cybersecurity is further emphasized by Brooks (2021), who points out that 78% of companies lack confidence in their cybersecurity regime, and only 5% of companies’ data are fully protected (Varonics, 2021). It is critical to align security awareness with the real and potential threats to organizations (Proofpoint, 2021).

The lack of security awareness exposes organizations to potential cyber threats and makes sensitive assets vulnerable to significant risk. Hackers tend to attack vulnerable employees of financial institutions, healthcare and manufacturing services. Remote or semi-remote working environments have made it more difficult for organizations to capture these attacks (Brandenburg & Paul, 2020). The health and financial industries are primary targets for malicious or criminal attacks (Tim, 2021). Ongoing cybersecurity awareness activities are required in organizations to address these dynamic threats. As such, this study aims to answer the following research question:

### **RQ**

What are the dimensions of cybersecurity awareness capabilities for an organization in a data-driven digital economy?

The core objective of Cybersecurity Awareness is to change individuals’ behavior so that they act appropriately in their handling of cyber threats (Alotaibi et al., 2016). Although several approaches, such as education and training programs, have been adopted to increase cybersecurity awareness, their focus is generally on understanding compliance issues, such as maintaining data confidentiality and reducing risk. While technical factors play a big role, human factors are also crucial, as a large number of internal data breaches are connected with human factors and issues that are either intentional, accidental, or with malicious intent. As a result, businesses cannot rely only on technological aspects of cybersecurity. Rather, an effective orchestration of organizational activities and management capabilities, along with technological infrastructure, is required on a continuing basis to effectively modify human behavior. While reconceptualizing cybersecurity awareness from a capability building perspective, it is also urgent to consider the data-driven aspects of the present economy. As exploring data-driven economic aspects has become an important research priority in general, explicating the microfoundations of CSA capabilities will benefit both researchers and practitioners in operations research.

## Literature review

### Cybersecurity

The key objective of cybersecurity for an organization is to protect that organization's data and information systems from cybercrimes through developing and adopting security controls and measures (Alqahtani & Erfani, 2021; Korpela, 2015). Cybercrime is defined as a purposeful attempt to jeopardize the valuable assets of an organization through a systematic effort to penetrate the organizational infrastructure (Maalem Lahcen et al., 2020). The attackers, either a group or lone individuals, attempt to identify potential vulnerabilities in a target organization (Bauer et al., 2017). The lack of robust and secured technological infrastructure, shortcomings in cybersecurity knowledge and experience of key personnel, the lack of education on cybersecurity protocols and compliance among employees, individual-level behavioural issues, or human errors may expose an organization and make them vulnerable to cybercrimes (Alqahtani & Erfani, 2021; Bauer et al., 2017). Based on a literature review of cybercrime trends and magnitude, Maalem Lahcen et al. (2020) shed light on the interdisciplinary framework related to human factors, behavioural, and decision-making strategies in cybersecurity. The authors find that technology alone cannot solve cybersecurity problems. We emphasize the importance of better understanding of cybersecurity in the workforce. Training and research for employees about the effectiveness of different approaches is necessary.

### Big data analytics and cybersecurity awareness

Currently, big data provides significant value to business organizations in its capacity to reveal and produce critical actionable insights of strategic importance about a business enterprise (Akter et al., 2020; Rawat et al., 2019). Through effectively applying big data and predictive analytics capabilities, organizations are now able to solve many critical business problems, as well as optimize business operations; these result in significant performance improvements (Akter et al., 2020). The present data-driven economy is producing an enormous amount of data, which is termed big data. Volume, velocity, veracity and variety are the four “V” key properties of big data that are emphasized by scholars (Rawat et al., 2019). Volume refers to the amount or size of the data being generated, velocity refers to the speed at which the data has been created, veracity refers to the data’s integrity or reliability, and finally, variety refers to the heterogeneity of the data being produced (Rawat et al., 2019).

The importance of data-driven cybersecurity has also recently gained the attention of scholars and practitioners (Rawat et al., 2019). Adoption of advanced analytics and visualization of data can make firms more vigilant, which can result in faster decision-making at the time of active cybersecurity threats (Böhm et al., 2018; Rawat et al., 2019). Hence, we can argue that relying on advanced technology and understanding the use of analytics and data visualization could be a dimension of cybersecurity awareness. Further, following human–computer interaction (HCI) techniques, it is possible to bridge the gap between individuals and their understanding of cybersecurity issues (Ki-Aries and Faily, 2017). Gaming applications that are tailored to users’ needs can help users effectively gain cybersecurity awareness. Even cybersecurity experts sometimes find descriptions of cybersecurity incidents complex and challenging to grasp in terms of their format or structure (Alotaibi et al., 2016). In the data-driven business era context, cybersecurity awareness requires careful consideration and implementation, following the unique nature of current challenges.

### Enabling cybersecurity awareness capability

Cybersecurity awareness is a state of consciousness in which users are fully aware of risk and security policies, supported by necessary knowledge and recognition of security threats. With CSA, users also understand the importance of being responsible. They act appropriately in relation to cybersecurity issues, and they abide by rules and regulations that are instituted by their organization’s security mission, by training programs in which they participate, and by regulatory bodies (Ahlan et al., 2015; Bauer & Bernroider, 2017; McCormac et al., 2017; Scholl et al., 2017). Cybersecurity awareness focuses on necessary skills to assist in safeguarding users from social engineering attacks. The process of social engineering takes place when an individual’s psychological properties are exploited with the intent to cause harm through a cyber-attack (Bitton et al., 2020). Kovačević and Radenković (2020) recommend considering security awareness as a continuous process due to the potential for new anticipated and unanticipated threats. An employee’s understanding of Cybersecurity Awareness Capabilities (CSAC) can positively influence their attitude towards cybersecurity compliance (Lee et al., 2016). Scholl et al. (2017) suggest that knowledge and actions secure and protect vital information as critical elements of CSAC. Gandhi (2017) defined cybersecurity awareness as the degree to which an individual is aware of cybersecurity, its conformity with policies and its commitment to the mission of an entity. Zhou et al. (2020) find empirical evidence that highlights the significant importance of psychological factors such as self-efficacy, risk awareness and social support to understand CSA in an individual, along with technical security embedded in a particular device or asset. Poepjes and Lane (2012) emphasize understanding how an individual acquires and manages awareness using individual capability when confronted with making a decision. Karjalainen et al. (2020) focus on behavioural changes of employees in dealing with cybersecurity over time and across different situations. Trim and Lee (2019) investigate the relevance of persuasive communication theory and motivation theory in facilitating cybersecurity awareness programs to influence changes in behaviors. The authors suggest further research in understanding the roles of managers in helping staff to fight cybersecurity attacks in a more effective manner.

Siponen (2001a, 2001b) explains various dimensions of cybersecurity awareness. The organizational dimension refers to managerial policies and activities related to cybersecurity awareness; the general public dimension explains how every citizen who is using IT should be aware of cyber threats; the socio-political dimension involves increasing knowledge among people who are working at a socio-political level, such as lawyers and politicians; the computer ethical dimension explains how scholars dealing with technologies should keep updated on their knowledge of cybersecurity. Finally, the institutional education dimension focuses on education and training at an institutional level. However, Sipoene (2001) states that due to the informal nature of cybersecurity awareness, there might not be any clear distinction between the microfoundations explained above. Granåsen and Andersson (2016) apply different technical performance measurement and behavioral assessment techniques based on real-life cyber defence scenarios to evaluate team effectiveness in a cybersecurity exercise among IT security experts. The study finds that cybersecurity awareness is necessary not only for cybersecurity personnel but also for operational managers. Employees need to be trained virtually on how to tackle cybersecurity threats while working remotely. These threats can originate from many actions and directions, from downloading a file onto a work computer to routine procedures to update a device.

At the same time, cybersecurity leaders must always be aware of new and evolving business environments. They must focus on more and new research and development, such as Wyse thin-client terminals, which allow all call staff to have secure remote connections (Anant et al., 2020). Table 1 reveals the scarcity of scholarly attempts to explicate different microfoundations of CSAC that are required to build and strengthen cybersecurity measures at the organizational level. More specifically, research providing comprehensive insights about CSAC is limited. To fill this critical gap, in the context of big data implications in business today, this study identifies CSAC as an organizational level capability and explicates the microfoundations of this important capability (Table 1).

**Table 1:** Seminal studies on Cybersecurity Awareness Capabilities (CSAC)

Study	Study type	Key findings on Cybersecurity Awareness Capabilities
Zwiling et al. (2020)	Empirical	The study aims to investigate knowledge and behavior on CSA regarding protection tools that are conducted in four countries, including Turkey, Poland, Slovenia and Israel. The study finds that cybersecurity knowledge is related to CSA despite differences in gender and geographical location. Further, the study reveals a significant difference of CSA knowledge and behavior across countries. The authors suggest that protective tools for cybersecurity management play an important role in creating awareness
Fabisiak & Hyla (2020)	Empirical	The study finds that Polish medical professionals lack mandatory knowledge about cybersecurity and need more training in this field. The authors reported difficulty in acquiring a large amount of data through surveys within the context of empirical enquiry
Bauer et al. (2017)	Empirical	Through analyzing efforts of information security managers’ in designing effective information security programs, the authors investigate how users perceive information security programs that lead to changes in behavior in maintaining information security compliance within the banking sector. The study finds that a comprehensive design strategy seems to be more effective among the bank’s employees for increasing cybersecurity awareness. The authors note that informants may convey biased information influenced by social desirability
Janabi & Shourbaji (2016)	Empirical	The empirical study reveals that the study participants, which include academic staff, university students and employees of universities, lack necessary knowledge and awareness regarding the implications of cybersecurity in their daily life. The authors suggest further research applying robust theoretical models and frameworks
Ahmad et al. (2018)	Empirical	Based on the data collected from mainstream schools (excluding private schools, international schools and special education), the study indicates that the level of knowledge on cybersecurity awareness among parents to protect their children from cybersecurity threats is moderate
Barth et al. (2019)	Empirical	The study tests the privacy paradox and finds that users are more concerned about ratings and the price of a desired application, than they are about privacy and security, when downloading and installing an app on their devices
Kraus et al. (2017)	Empirical	The study is conducted on job seekers and students and finds that security and privacy actions on the devices of smartphone users are influenced by intrinsic motivation by nonessential psychological needs, such as the need for security along with other needs
Shanfari et al. (2020)	Empirical	The study finds a significant impact of six independent variables that may affect human components in adopting CSA, considered in the empirical context of Oman’s public sector employees
David et al. (2020)	Empirical	The authors find that human beliefs, consisting of resource belief, usefulness belief, and reciprocity belief, have a positive correlation for gaining specialist knowledge in cybersecurity
Bavel et al. (2019)	Empirical	The study applies protection motivation theory to investigate changes of user’s online security behavior through providing notifications during online shopping. The findings suggest that factors such as awareness of cybersecurity measures, risk attitude, age, and country have an impact on appropriate protective behavioral response
Tschakert and Ngamsuriyaroj (2019)	Empirical	The authors find the impact of classroom training with respect to phishing emails and reducing vulnerability among the participants to be insignificant when compared with users who do not receive any training whatsoever. The authors recommend further investigation on the usefulness of the measures, and suggest that educating participants about the study may itself sensitize participants toward phishing and cybersecurity learnings
Schneider et al. (2020)	Empirical	Based on a literature review and in-depth interviews with cybersecurity experts and senior managers, this study offers a managerial information security awareness guideline that is proven based on outcomes. To assess a practicable managerial information security awareness program, the authors recommend targeting senior managers in their specific and desired environment
Holdworth & Apeh (2017)	Empirical	The study finds that to be successful, industry requirements for designing and implementing structured programs and training for establishing cybersecurity awareness among hospitality industry employees, needs to involve three stages of artefact evaluation. The authors suggest that a greater number of interactive elements are necessary for the programs
Zuopeng & Zhang (2019)	Empirical	Through reviewing online content such as blogs of corporate websites, the study provides actionable guidelines for the successful implementation of cybersecurity training and awareness programs within an organization

## Methods

This study explores the dimensions of cybersecurity awareness capabilities using a systematic literature review (SLR). The nature of systematic literature review is to minimize bias by means of extensive literature review to explore important scientific contributions within a field and poses a question (Tranfield et al., 2003). A systematic literature review helps us to understand if a result is persistent throughout the studies and to find out what future research is needed. Although not all studies might fit under the approach of systematic review, it is considered the most appropriate and meticulous approach for reviewing articles to cover all applicable data (Snyder, 2019).

Some papers published in journals have flaws. They may fail to include/exclude proper articles; their methodologies may not be backed by proper discussion or may lack critical assessment. On the other hand, a systematic approach tends to extensively track down and incorporate studies that revolve around specific questions (Palmatier, 2018). Therefore we have chosen the systematic literature review to address our research question on the dimensions of cyber security awareness capabilities.

An example of a systematic review in cybersecurity research is a study conducted by (Gheyas and Abdallah 2016). The study conducted a systematic review of over thirty-seven studies of peer-reviewed journals, edited books and conference proceedings to address two questions related to insider threats. Furthermore, studies from the following paper-reviewed journals (e.g., Taylor et al., 2020; Spanos and Angelis 2016) have used the systematic literature review to address cyber security concerns in different fields such as education, the stock market, blockchain, and private and public organizations.

Following the guidelines of established SLR research in operations (e.g., Akter et al., 2020) and reference disciplines (e.g., Palmatier et al., 2018; Snyder, 2019; Tranfield et al., 2003), the current study explored the most relevant databases, such as *ABI/Inform Collection (ProQuest), Business Source Complete (EBSCO), ScienceDirect, Emerald Insight, Wall Street Journal (ProQuest).* We appliedvarious relevant search strings to address our research questions, namely “cybersecurity”, “cybersecurity awareness”, “cybersecurity awareness capability”, “management capability”, “technology capability”, “data governance capability”, “cybersecurity knowledge”, “cybersecurity training,” etc. After screening the title, abstract, manuscript and keywords, we identified 57 papers from the initial identification of 307 articles. We added 5 more papers from cross-citations, which resulted in a total of 62 articles.

At this stage, we conducted a thematic analysis using the procedures of Braun and Clarke (2006). The findings presented us three primary dimensions (i.e., personnel, management, infrastructure capabilities) and eight subdimensions (i.e., knowledge, attitude, learning, training, strategic orientation, technology and data). We confirmed the reliability of the themes through qualitative analysis of the data using QSR NVivo 12. These themes were further confirmed by a panel of five judges (3 academics + 2 practitioners) using a Q-sorting procedure with a nominal scale of 1 = personnel capabilities, 2 = management capabilities and 3 = infrastructure capabilities). The inter-rater reliability of the themes was checked by applying IBM SPSS statistics package (version 26) (Akter & Wamba, 2016) with a score of 0.86 Kalpha (De Swert, 2012; Krippendorff, 2004, 2007).

Thematic analysis can be defined as a procedure for pinpointing, analyzing and describing themes within data. The advantage of using thematic analysis is that it can be used for interpreting data and is well suited for qualitative analysis, for example, in policy developmet. The thematic approach can construct an insightful analysis that helps to answer research questions (Braun and Clarke 2006). It is considered the most relevant technique for research that seeks results through interpretation. There are several studies in the domain of cybersecurity that uses a thematic analysis approach. For example, a study conducted by (Liu et al., 2020) interviewed thirty-six professionals and used thematic analyses to find factors that lead to cyber risk in Connected and Autonomous Vehicles. The study identified six factors, including awareness, user and vendor education, responsibility, and trust. Similarly, a study conducted by (Cains et al. 2021) used thematic analysis to address research questions which led to determining themes.

## Theory

Dynamic capability view (DCV) offers a theoretical underpinning to transform organizational resources and capabilities according to changes in the external environment. To effectively address changes in the external environment, DCV scholars recommended transforming the organizational resource base (Eisenhardt & Martin, 2000); sensing, seizing, and re-configuring organizational resources and capabilities (Teece, 2009; Teece et al., 1997); reconfiguring organizational learning patterns (Zollo & Winter, 2002); and finally, simultaneously pursuing exploration and exploitation (Smith & Tushman, 2005). Within the context of the volatility, uncertainty, complexity and ambiguity of the digital business environment, DCV offers normative guidelines to managers to formulate appropriate strategic courses of action to navigate their organizations in an entrepreneurial, innovative and ambidextrous manner (Raisch et al., 2009; Schoemaker et al., 2018).

At the individual level, CSA is articulated as an individual employee’s psychological and behavioral capacity for awareness of the importance of cybersecurity for committing to normative rules, policies, and guidelines to act effectively towards potential security threats following the cybersecurity mission of an entity (Ahlan et al., 2015; Gandhi, 2017; McCormac et al., 2017; Scholl et al., 2017). Security workforce capability is the combined expression of security proficiencies in knowledge and practical skills of an individual for implementing appropriate actions, reactions or inactions for successful security of the operational system (Ani et al., 2019). At the organizational level, CSAC can be considered an ongoing process following systematic methods to enable effective preventive, proactive and reactive measures against perceived cybersecurity threats through the fostering of necessary psychological capabilities and human capital among employees (Bada et al., 2019; Bitton et al., 2020; Kovačević & Radenković, 2020). Therefore, fostering CSAC across organizational boundaries means emphasizing diverse managerial skills and capabilities in order to harness benefits for the current digital infrastructure facing extensive security risks (Bitton et al., 2020).

CSAC is a purposeful accomplishment requiring the orchestration of an individual’s routines and resources across organizational boundaries to result in an intended outcome in a predictable and systematic manner (Barney, 1991; Barney & Felin, 2013). Further, CSAC aims to inform and modify individual and organizational level capabilities as well as the technical infrastructure, under external changes. External changes may include technological changes and changes in the capabilities of cybercriminals or threats (Al-Shanfari et al., 2020; Holdsworth & Apeh, 2017; Granåsen & Andersson, 2016). Due to the external orientation of cyber threats and the dynamic role of CSAC to sense, seize and reconfigure organizational resources, capabilities, or learning (Teece, 2009; Zollo & Winter, 2002), CSAC can be considered as a dynamic capability.

The importance of managerial roles in building dynamic capabilities is documented in extant literature (Adner & Helfat, 2003; Gavetti & Levinthal, 2000; Helfat & Peteraf, 2015; Martin, 2011; Sirmon & Hitt, 2009). Teece (2009) highlights the vital roles of managers in facilitating strategic decisions through nurturing supporting culture, articulating goals, fostering trust and facilitating organizations to take rapid actions related to opportunities and threats arising in the external environment. Managerial skills and capabilities play particularly vital roles in addressing challenges arising in the external environment related to ongoing cybersecurity threats that result from rapid technological advancement. CSAC, managerial awareness, perception, and prompt identification of potential cybersecurity threats demand superior cognitive capacity of individual employees (Gandhi, 2017; Kovacevic et al., 2020). Further, individual employees’ knowledge and learning about cybersecurity threats in a practical manner enable them to respond to cyber threats faster (Bohm et al., 2018; Bauer et al., 2017; Ani et al., 2019). Managers need to facilitate communication and dialogue across all levels of organizational structure (Salvato & Vassolo, 2017) to build a dynamic community that also supports weak performers (Eisenhardt & Martin, 2000). This point is echoed by Li et al. (2016) in the cybersecurity context. The authors recommend fostering a culture of knowledge sharing among employees to improve knowledge and awareness of cybersecurity. They highlight the significance of diverse socio-political backgrounds among employees, which can lead to a positive influence with improved perception of potential cybersecurity threats (Siponen, 2001a, 2001b).

## Conceptual model

**Fig. 1:**
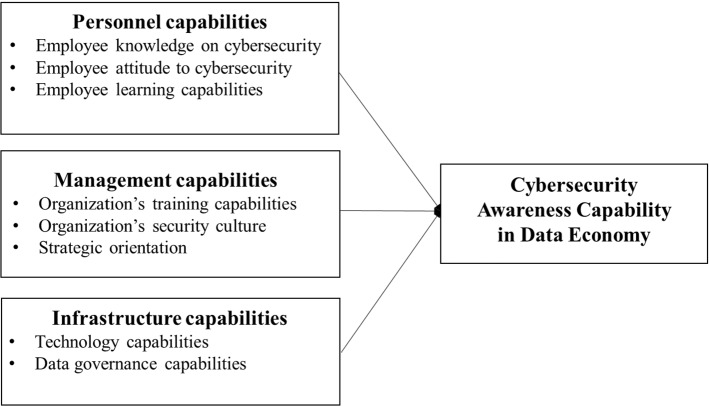
Microfoundations of data-driven cybersecurity awareness capability

The findings of our review identify CSAC as an emerging research domain in business and management research, but one that has very limited research in this particular stream. Although there is a paucity of research on CSAC, our findings identify three major dimensions and eight subdimensions of CSAC. Figure 1 presents the necessary microfoundations to build organizational level CSAC across personnel, management and infrastructure capabilities. To gain organizational business value out of CSAC, firms first need to develop their personnel with knowledge, attitude and education. Second, firms need to manage proper training, organizational culture and strategic orientation. Finally, firms need to establish the right infrastructure with data governance and technology to build CSAC.

### Personnel capabilities

Employee knowledge and attitudes are critical to reducing cybersecurity threats (Wahyudiwan et al., 2017). We argue that the knowledge, attitude and behavior (KAB) model of awareness can be used to measure the CSA level of an employee (Wahyudiwan et al., 2017).

#### Employee knowledge of cybersecurity

Employee knowledge of cybersecurity has a significant impact on building CSAC in the data-driven business context. The level of security knowledge is defined as how much knowledge an individual possesses of theoretical information on cyber threats, vulnerabilities, patterns of attacks, and the impact these can have on the system (Ani et al., 2019). Empirical findings have shown that computer literacy skills and information seeking skills also can affect an individual’s behavior in handling security (Anwar et al., 2017). Safa and Solms (2016) emphasize the importance of knowledge sharing to reduce threats as well as costs for information security.

#### Employee attitude towards cybersecurity

An employee’s attitude can be described as a negative or a positive feeling towards a behavior. Attitudinal influences can arise from factors such as job performance or workgroup norms (Gaspie et al., 2017). Due to employees’ tendencies to neglect policies, training and education on cybersecurity, and lack of initiatives by companies to pay necessary attention to this issue, users in an organization often fall victim to cybersecurity threats by breaching policies unintentionally or as a result of influence by other external factors (Ahlan et al., 2015). Individual factors (self-attitude, self-behavior and self-cognition), institutional factors (policy compliance and training programs) and environmental factors (peer performance, social pressure, perceived threats and religious indicators) provide a deeper understanding for measuring CSA, especially for a knowledge-based institution (Zwilling et al., 2020). Based on empirical findings, Li et al. (2016) suggest that the influence from peer behavior and other employees’ actions can play a vital role in improving cybersecurity behavior in an organization. Therefore, necessary measures should be taken, such as providing rewards to employees who help create a pro-security atmosphere, to create a positive effect.

Several social, psychological and demographic factors such as age, gender, etc., can impact an employee’s behavior in handling cyber threats. Previously, protection motivation theory has been applied to explain why different genders in a working environment tend to behave differently on handling cyber threats. However, recent studies have shown that security behavior is correlated with variables such as perceived susceptibility, perceived severity, perceived benefits, and self-efficacy (Anwar et al., 2017). The attitude of employees toward a cyber threat is often ignored given the difficulty of assessing an individual’s awareness levels and whether or not they possess adequate skills to respond to cyber threats (Hadlington, 2018).

#### Employee learning capabilities

Employees that exhibit reluctance to comply with cybersecurity measures in an organization may cause serious threats. Therefore, top management needs to take action to effectively educate employees about cyber threats by providing instructions that are easy to understand (Siponen et al., 2014). Horenbeeck (2017) recommends keeping the rules simple for employees to better understand security threats and policies, as complex security rules often lead people to take shortcuts. A survey based on behavioral theory showed that an organization needs to put social pressure on employees from superiors and peers. In this case, managers play a vital role in instituting effective policies and educating employees about the seriousness and devastating effects of security threats on the organization (Siponen et al., 2010).

### Management capabilities

#### Training capabilities

To enhance employees’ capability for recognizing cyber threats, organizations need to invest in training (He & Zang, 2019). However, due to employees’ lack of interest and past experiences feeling bored in the training program, organizations face difficulties in reaching goals to educate their employees. Siponen et al. (2010) note that lack of motivation and enthusiasm is also a strong indicator of failure for training programs. Further, employees also complain about a lack of specificity and the generic nature of training materials that lack consideration of varied responsibilities and different levels of security threats faced by individual employees. The head of IT security of Nominet UK believes that if employers offer more rounded and holistic training and take into account security incidences involving employees and reflect on actions taken, that such changed approaches will benefit both employees and the organization (Caldwell, 2016). Finally, security programs must be updated both when threats change and when there are technological advancements (Siponen et al., 2010).

The goals of programs should not only be to create awareness but also to provide training about what to do when faced with a threat. Hence, practical methods should be used, such as when a suspicious email arrives, the email can be forwarded to a security expert rather than ignoring it. The principal security researcher of Kaspersky Lab concluded that the main goal of the training should be to develop a security mindset for the employees so that they can take action when they encounter a threat (Caldwell, 2016).

In terms of training cybersecurity personnel, an exercise training program known as cyber defence exercise (CDXs), has proven to be useful as the goal of CDXs is to provide interactive training with real-life scenarios in a controlled environment. The program can also be useful in other work areas, such as legal and forensic work (Granåsen et al., 2019). Experts believe that awareness training programs should be available to employees regularly through various mechanisms, such as SMS, via email, using office floorwalkers, or through hackathon events.

To develop effective training content, it is critical to understand employee behavior related to online security. Companies often prefer developing strict policies and installing cutting edge technologies for security. However, inappropriate human behavior alone can expose the company to danger (Li et al., 2014). A global phishing survey has shown that phishing attacks usually target e-commerce, banks and money transfer industries. Phishing often involves stealing sensitive consumer information and observing victim’s behavior (Arachchilage et al., 2016). Research findings demonstrate that users’ behavior can be changed through phishing education, resulting in increased awareness capabilities of phishing threats. However, to effectively modify an individual’s behavior towards cybersecurity threats, training is necessary but may not be sufficient. In addition to training, simulation of attacks with real-world examples and immersive programs can add value.

To be effective, the trainer conducting the training needs to be very knowledgeable and should possess a sound capacity to deliver the content. Further, the mode of delivery of the training is important as recently it has been shown that mixed learning methods consisting of both online and face to face learning deliver better results for creating awareness. Training and learning tools such as the Cyber Defense Exercise (CDE) often identify an increasing gap between training methods and technological progression. One of the key factors for a successful cybersecurity training program is the incorporation of human factors such as decision-making skills, negotiating skills and information-sharing capabilities. Further, an individual should be provided training on cybersecurity from the earliest stage of employment, with effective training reflecting practical scenarios to attain mental readiness during unexpected incidents (Knox et al., 2019).

On the other hand, insider threats (deliberate or accidental) are a growing concern, and several security experts believe that general training fails to recognize the issue of internal threats. Unfortunately, most often, organizations do not take internal breaches seriously until there is data leakage or brand damage. Therefore, training employees about internal threats has to be taken seriously before data falls into the wrong hands (Caldwell, 2016), but more importantly, the right policies need to be in place regarding acceptable company practices.

#### Security culture

An organizational security culture can be defined as a collection of shared security values, assumptions and beliefs of cybersecurity in an organization that can shape employee behavior (Chen et al., 2015). Cybersecurity culture can be viewed as a sub-culture of an entity with specific goals of security, including all socio-cultural and technical measures. Security culture influences employees to have a security mindset and commensurate behavior. Prior studies have shown that there is a clear link between establishing security policies and the influence of top managers in building a security culture (Chen et al., 2015). Several countries, such as UK, US, Canada and South Africa, identify cybersecurity culture as a critical element of an organizational policy framework (Gcaza et al., 2017).

To establish a proper cybersecurity culture, management must have a vision and a strategy with appropriate policies and procedures to change the security culture in an organization (Da Veiga, 2016). Alshaikh (2020) provided an analysis of how three Australian organizations have improved their cybersecurity culture through five key initiatives: first, to identify key cybersecurity behavior; second, to establish a cybersecurity champion network; third, to build a cybersecurity hub by creating a learning environment for the employees; fourth, to develop a brand for the cyber team to make it more visible; finally, fifth, to align security awareness activities with internal awareness programs and external cybersecurity campaigns. An organization’s security culture is impacted by the positive attitude of employees who follow cybersecurity compliance guidelines (Gaspie et al., 2017).

#### Strategic orientation

Rapid technological change forces frequent updates of the necessary skillsets for security managers; therefore, the most important and updated skill sets must be identified along with a proper way of delivering them to the professionals. Cybersecurity managers require a certain set of skills to better understand and manage information security. It is vital for cybersecurity experts to be aware of the capabilities that are required for understanding and addressing cyber threats in an organization (Haqaf & Koyuncu, 2018). Despite the availability of information about the necessary skillsets for cybersecurity managers, gaps are still present. Haqaf and Koyuncu (2018) further depict the importance of assessing what skill sets are required in the changing cybersecurity environment. Nazareth and Choi (2015) state that cybersecurity managers have several important responsibilities and functions, including security planning, managing risk, selecting proper technology, assessing threats, formulating policies, monitoring performance, and implementing counter measurements and maintenance.

Managers play a vital role in running organizations; therefore, it is necessary for managers to understand not only the threats that can arise from a technical perspective but also those that grow out of human behavior. Understanding bothtechnical and human factors can help to mitigate threats; hence, a management success factor (MSF) model can help decision-makers in an organization deal with cybersecurity threats more efficiently (Diesch et al., 2020). An MSF model’s main purpose is to identify possible factors or elements that can be used to make better decisions (Diesch et al., 2020). Further, Nazareth and Choi (2015) developed a model that allows cybersecurity managers to make better decisions on an organization’s information assets, and the model additionally provides security managers with clear instructions about the kinds of investments that are needed and the impacts those investments can have. Security managers often use strategies that include detection, deterrence, vulnerability reduction, education and training. However, clear strategic steps are required rather than approaching with a single solution.

Recently, network breaches have become so common that only the most significant breaches make news headlines, such as the breach of the credit reporting company Equifax Inc, which affected over 143 million consumers (Kolevski et al., 2021; Rothrock et al., 2018). Boards of directors play a vital role in providing cybersecurity to a company; however, a study showed that most boards are unprepared to handle cybersecurity threats despite 58% of board members believing cyber-related risk is the most challenging risk they expect to oversee. The importance of the top executives and the role of board members is exemplified by the data breach of Target Corp in 2013, where the personal information of over 60 million customers was stolen. The shareholders took legal action towards the company, which ultimately caused the CEO and the CIO of the organization to resign. A study conducted by Rothrock et al. (2018) shows that senior executives and board members are not asking the right questions because they do not have meaningful metrics to evaluate cybersecurity issues related to their businesses. Deloitte (2015) shows that audit committees should increase their interactions with the IT department in order to better understand cybersecurity threats; technology experts should also join board meetings in different organizations to raise awareness among the members. Overall, an organization’s strategic direction can shape its awareness capability.

### Infrastructure capabilities

#### Technology

Organizations must keep their operating systems up to date to minimize threats. An example can be taken from the cyber attack of 12th May, 2017. Ehrenfeld (2017), depicts that the attack, used WannaCrypt and targeted Microsoft Windows across 150 countries. The attack infected computer systems of various sectors, including transportation, energy and healthcare. Britain’s National Health Service stated that their systems and machines all were impacted. Two days later, Microsoft came up with a solution, but it took over 50 days to apply the solution properly after many failed attempts. From these examples alone, we can see the danger of cybersecurity breaches and the importance of staying up-to-date with the latest technology in order to exhibit equal strength with cyber-attackers.

Due to recent technical advances, the healthcare system uses equipment that is connected with other networks and devices, leaving the overall systems vulnerable, including medical devices. The U.S. Food and Drug Administration (FDA) has responsibility for assuring the safety of medical devices, and they have acknowledged the seriousness of the problem. However, medical device manufacturers seem to have neglected the importance of providing security for data transfers and data storage. The exchange of data and the collection of data that supports clinical decision making is not only vulnerable to a medical device’s characteristics and connectivity, but technological issues, software risks, and of course, human factors also seem to play a vital role (William and Woodward, 2015). Kim (2017) states that cyber-attacks can cost government organizations thousands to millions of dollars. For example, Telnet is still used in organizations, which is very outdated and leaves an open door for an attack. More up to date technologies and tools are required. But these issues are often overlooked.

In organizations’ efforts to maintain updated technology in cybersecurity, emerging technologies such as data analytics, machine learning, artificial intelligence and blockchain technologies have proven to be very important to managing cybersecurity. New technologies such as quantum computing, cloud computing, predictive semantics, behavioral identity, and dynamic networks will bring new approaches for improving cybersecurity but will also create new cybersecurity threats (Geluvara et al., 2019). Traditional data-driven technology solutions have drawbacks, such as inefficiencies in storage, retention, access, and processing of the large volumes of information produced by big data (Rawat et al., 2019). As these techniques were not designed to handle semi-structured or unstructured data, the challenges of traditional tools can be addressed by big data technology (Rawat et al., 2019). But these same technologies, e.g. artificial intelligence, can be used skillfully by attackers to penetrate cybersecurity barriers through offensive machine-level ‘learning by doing’ approaches.

Machine learning (ML) offers superiority over traditional rule-based algorithms, and ML methods are now being used to enhance cyber-security capabilities. Techniques can be applied for detecting intrusion, malware and spam. We need to assess the solution that is provided by ML and find its limitations as well (Apruzzese et al., 2018). Detecting a new generation of malware and cyber threats tends to be difficult with traditional cybersecurity procedures, which include access control, antivirus and cryptographic software, intrusion detection and prevention systems, sandboxes, etc. Therefore, solutions to the problem rely on ML and artificial intelligence (AI), which can rely upon data from earlier attacks and respond to newer ones. AI is used in cybersecurity for faster detection of threats and attacks in a given situation. A further example has been illustrated by Geluvara et al. (2019) on how AL, ML, and DL have helped fight real cybersecurity problems, for example, London’s NHS spotted an attack within a second using their algorithms, and the threat was eliminated without any damage. Similarly, the MIT Computer Science and AI Lab successfully built a model that was capable of filtering millions of data points and passing the results to a human analyst; AI was also utilized by the companies PatterEx and CSAIL, who then found that their attack detection rate rose by 85%.

Blockchain technology has recently gained significant adoption across business ecosystems. Blockchain provides trusted transactions among participants in a network. The uniqueness of blockchain technology has opened doors for many industries such as logistics, banking and pharmaceuticals in the context of cybersecurity. Blockchain has the potential to enable a new breed of decentralized applications that will not require any intermediaries for building key elements of cybersecurity infrastructure (Taylor et al., 2020). We suggest that the use of blockchain or related technologies does not provide a silver bullet for cybersecurity issues, but the technology does provide support to existing systems in IoT, data storage and sharing, network security, private user data, navigation, and the utility of the World Wide Web.

Several organizations and businesses are now embracing the service of Security Operation Centers (SOCs). SOCs can be defined as a centralized location, within or outside of an entity, consisting of people, technologies and processes with an aim to provide complete cybersecurity solutions, including awareness, maintaining compliances and threat management (Agyepong et al., 2020). Many organizations that cannot afford SOCs have had to rely on a third-party security provider, which is often referred to as a Managed Security Service Provider (MSSP) (Agyepong et al., 2020). Mutemwa et al. (2018) describe the tools that are used by SOCs. First are security information and event management systems (SIEM) tool that looks at events statistically from various network sources such as hosts, the network endpoint, and servers. The SIEM tool provides a risk analysis procedure by analyzing log data. Second are threat intelligence tools, which gather threat intelligence from various sources such as news, social media and the centralized database of an organization. The third is vulnerability assessment, with an investigative and forensic tool. These are tools that help assess websites and operating systems. Finally, there is a storage tool. All SOC tools should be protected and encrypted so that only authorized personnel can access them.

#### Data governance

Data is precious to organizations, and protecting data has become more important than ever before. Thus, data governance plays a vital role in helping organizations understand what kind of data they must protect. Data governance (DG) can be defined as the processes, procedures, technologies and people that enable an organization to exploit data as a digital asset (Yang et al., 2019). DG provides a general framework for maintenance and administration of data security, availability, quality, usability, integrity and relevancy. DG also helps organizations set business goals, maintain business processes, and make complex decisions. DG practices involve a guided framework for collecting, managing, storing and utilizing data (Yang et al., 2019). Organizations may face serious challenges for data governance such as lack of a big data governance framework, shortage of skilled labor, big data security and privacy, lack of required tools for generating insight, organizations capability to understand the use of data, insufficient knowledge of managers, organizational capability for digital transformation, and the complexity of data collection and storage (Rawat et al., 2019; Yang et al., 2019).

It is important to distinguish between governance and management. Whereas governance refers to those who make the decision for ensuring effective management and use of resources, management involves the implementation of the decisions made by governance (Alhassan et al., 2016). Thuraisingham (2019) states that corporate executives and the governing board must ensure cyber governance in an organization, which includes activities such as data privacy, carrying out risk analysis, and protection from cybersecurity threats. The board and executive members must go through a few steps to properly implement cyber governance, such as having a cybersecurity security expert among the board members, having in-house cybersecurity experts, or have someone from a reliable and reputable source provide these services. Eugen and Petruţ (2018) illustrate management practices for protecting data and recommend having a risk based approach towards security, creating hierarchical cybersecurity policies, maintaining updates and security patches, testing and accomplishing backups, handling passwords securely, having physical security measures, educating users, employing tools for monitoring, analytics and management, implementing a comprehensive endpoint security solution, and providing network security devices. They further state that if an organization’s data is compromised, it may reduce an organization’s capability to provide services, eventually leading to fraud, disclosure of confidential information, or destruction of data.

## Discussion

The microfoundation perspective (Barney & Felin, 2013; Felin, 2015) provides a reductionist view of the underlying elements of CSAC, which will pave the way for building a holistic view on CSAC by providing deeper insights into the effectiveness of cybersecurity measures and programs. We have seen many organizations with superior CSAC. However, concern still remains about the sustainability of these programs, and an in-depth study has still not been conducted to date to deal with the CSA issues in an organization and to provide an effective solution.

### Theoretical contributions

This study makes several theoretical contributions. The study extends the dynamic capability view (Eisenhardt & Martin, 2000; Schoemaker et al., 2018; Schoemaker et al., 2018; Smith & Tushman, 2005; Teece et al., 1997; Zollo & Winter, 2002) through applying this essential theoretical perspective to the field by introducing a new dynamic capability, namely CSAC, which consider the context of data driven business ecosystems. Previously, scholars have investigated CSA following criminological theories such as the general deterrence theory (GDT) or limited versions of GDT such as the theory of reasoned action, protection motivation theory, theory of planned behavior and also psychological theories such as protection motivation theory or situational theory (Hanus Windsor & Wu, 2018). As such, this study is the first study based on DCV applied to cybersecurity awareness. The identified microfoundations for CSAC highlight the importance of transforming individual level and management level capability and organizational infrastructure for the successful development of CSAC, resulting in superior information and cybersecurity management performance.

Firstly, following DCV, the findings of this study extend the understanding of managerial roles in building CSAC (Adner & Helfat, 2003; Helfat & Peteraf, 2015). Extant studies on managerial roles in building dynamic capabilities have recognized the important relationship between quality of managerial decisions, strategic changes and firm performance (Helfat & Martin, 2015; Martin & Bachrach, 2018), and organizational capacity to change and maintain superior performance (Widianto et al., 2021). The theoretical underpinning of dynamic capabilities, therefore, will be of importance in cybersecurity issues, which are a serious concern among managers in the present rapidly changing business context. Extending dynamic capability theory (Adner & Helfat, 2003; Helfat & Peteraf, 2015) within the context of cybersecurity will allow researchers to integrate the normative guidelines of the dynamic capability view into the context of cybersecurity in an effective manner.

Secondly, this paper demonstrates the necessity of transforming and reconfiguring organizational resources and capabilities, including individual behaviors, training and learning methods, and technological infrastructure in accordance with the changes in the external environment through upgrading and integration of new technologies. As DCV endeavors to equip managers with appropriate courses of action to tackle the challenges posed by the rapidly changing external environment (Raisch et al., 2009; Schoemaker et al., 2018; Teece, 2009), Akter et al. (2020) echo that technological advancement fostered by next-generation technologies creates serious challenges for managers of businesses across different industries and sectors. Conceptualizing CSAC as a dynamic capability along with its underlying microfoundations offers a sound theoretical underpinning to comprehensively elaborate the individual and management capabilities and technological infrastructure necessary to tackle increasing cyber threats to present-day business organizations. This study extends the theoretical movement of microfoundations in DCV (Barney & Felin, 2013; Felin, 2015). The conceptual model and findings of this study shed light on the microfoundations of the CSAC, which will extend the interactions among various organizational factors and functional elements to carry out effective cybersecurity awareness programs.

### Practical contributions

Increased cyber-attacks have affected business organizations. There is an increased risk of loss of sensitive strategic information, customer’s information, and valuable assets (Agilient, 2019). The negative consequences of cyber threats to business organizations include but are not limited to destruction and damage of proprietary and commercially sensitive transactional and personal data, loss of financial assets, risk of loss of intellectual properties, including post-attack damages, include the cost of restoration and recovery procedure as well as the loss of trust and reputational harm (Morgan, 2020). Further, the global pandemic has caused companies both large and small to shift toward a remote working environment. Therefore, organizations must understand and have the capacity to deal with the risks of working remotely. The pandemic made the workforce more distracted from cyber threats, and to make matters worse, security professionals also had issues with working remotely. Hence companies must prioritize cybersecurity budgets and investments and assess risk accordingly.

Training and exercises are needed to address these changes. Since the pandemic began in the USA alone, the FBI has received over 4000 complaints daily from different corporations. As human error still makes up 90% of all data breach cases, measures should be taken to herald changes in training and exercises. Employees must be reminded about their role on how to effectively prevent, detect, respond and recover from cyber-attacks. Management should provide new guidelines and monitor the success of employee training and learning activities, role-based training programs, and exercises to raise and strengthen awareness. These programs must be considered for every level of employee (Brandenburg & Paul, 2020).

Our conceptual framework based on an extensive analysis of prior work suggests that developing CSAC involves the dynamic involvement and intertwined contribution of personnel, management, and infrastructure. For example, prior research highlights that the development of organizational capabilities stems from the capacities of individuals (Teece, 2007, Felin et al., 2012; Foss, 2011). Accordingly, the CSA level of employees, as determined by their cybersecurity knowledge, attitude, and learning behavior, is critical in forming the basis of CSA management capabilities, such as an organization’s CSA climate, or the shared perceptions of individuals within the organization around the importance of risk and security policies, and knowledge and recognition of security threats. In turn, with a concerted focus and emphasis on CSA (e.g., the organization’s CSA orientation), the organization will be in a better position to develop the necessary organizational processes and routines for the continuous renewal and reconfiguration of data governance and cybersecurity technology (i.e., infrastructure capabilities). Our illustration here emphasizes the joint importance of the micro-foundations of personnel, management, and infrastructure in developing CSAC.

Similarly, scholars have suggested that resources at the top management level influence how managerial decisions are made, and how they affect organizational operations and outcomes (Helfat & Martin, 2015; Bendig et al., 2018). Accordingly, the strategic infrastructure firms deploy to manage cybersecurity threats (e.g., technology and data governance capabilities) are influential in shaping the cybersecurity knowledge, attitude, and learning behavior of individual employees (e.g., personnel capabilities) because the infrastructure reflects the firms’ organization-wide values and beliefs, as well as operational activities, on embracing and espousing CSA (e.g., the firms’ CSA strategic orientation, culture, and training capabilities). Therefore, in developing CSAC, the micro-foundations of personnel, management, and infrastructure capabilities should not be considered in isolation. Instead, managers are advised of the important roles jointly played by personnel, management, and infrastructure capabilities underpinning their continuous organization-wide emphasis on cybersecurity.

## Future research and conclusions

In developing our CSAC framework, we synthesized diverse literature streams that somewhat overlap or are partly grounded in diverse assumptions. Although our CSAC framework is specifically geared toward specific organizational settings, it may also apply to dyadic interactions, particularly those characterized by collaborative roles (e.g., within a supply chain). However, we do not contend that all micro-foundations will always be equally important or that high CSAC will always be required to realize desired outcomes. Some micro-foundational elements (e.g., infrastructure capabilities) may be more pertinent in some contexts (e.g., when collaborating with external partners) compared to others (e.g., when interacting with end-users, where personnel capabilities might be of greater importance).

Appropriately, empirical research is necessary to corroborate our CSAC concept and model. Grounded in dynamic capability theory, our conceptualization of CSAC emphasizes the continuous (re)configuration of organizational resources and capabilities to not only sense but also combat cybersecurity threats in an ongoing manner. Future research may adopt a longitudinal approach to examine the extent to which the fundamental micro-foundational elements identified in our conceptual framework contribute to firms’ continuous development and deployment of CSAC. Future research may also benefit from employing the same approach to determine the extent to which firms’ CSAC facilitate different performance outcomes, such as cybersecurity breaches or operational efficiency.

Given our conceptualization of CSAC as spanning across multiple organizational levels (e.g., individual and firm), future research may be able to shed further light on the specific processes and mechanisms through which firms develop CSAC. For example, previous research highlights the importance of individual behaviors as the fundamental building blocks of organizational capabilities at the firm level (e.g., Wahyudiwan et al., 2017). Accordingly, a multi-level approach could be employed to examine the extent to which personnel capabilities fostered at the individual level contribute to CSAC at the firm level.

Future research may also benefit from incorporating relevant boundary conditions to examine the specific contexts under which the role of personnel, management, and/or infrastructure capabilities are more or less pronounced in facilitating the development and deployment of CSAC. For example, recent research finds that the accelerating rate of digital transformation on economic performance is quicker under low market turbulence, but results in worse environmental performance when market turbulence is high (Li, 2022). Accordingly, given dynamic capability theory emphasizes that firms should develop processes for resource reconfiguration and capability enhancement to evolve and fit with changing market conditions (Teece, 2007; Morgan, 2012), it might be insightful to investigate which specific CSAC micro-foundation is more or less relevant in what specific industry condition (e.g., technological turbulence or market turbulence).
